# Bone Health and Natural Products- An Insight

**DOI:** 10.3389/fphar.2018.00981

**Published:** 2018-09-19

**Authors:** Vasanti Suvarna, Megha Sarkar, Pramila Chaubey, Tabassum Khan, Atul Sherje, Kavitkumar Patel, Bhushan Dravyakar

**Affiliations:** SVKM’s Dr. Bhanuben Nanavati College of Pharmacy, Mumbai, India

**Keywords:** phytochemicals, osteoporosis, bone disorders, osteoclast, osteoblast

## Abstract

Bone metabolism involves a complex balance between matrix deposition, mineralization, and resorption. Numerous evidences have revealed that dietary components and phytoconstituents can influence these processes, through inhibition of bone resorption, thus exhibiting beneficial effects on the skeleton. Various traditional herbal formulae in ayurvedic and Chinese medicine have shown demonstrable benefits in pharmacological models of osteoporosis. The present review discusses normal bone metabolism and disorders caused by bone disruption, with particular reference to osteoporosis and current therapeutic treatment. Furthermore the effects of constituents from natural products on bone tissue are explained, with relevant evidences of efficacy in various experimental models.

## Introduction

Osteoporosis has become a global health issue of major concern. It is defined as a condition illustrated by micro-architectural deterioration and low mineral density of bone tissues resulting into enhanced bone fragility and increased risk of fracture. Osteoporosis shows its existence in several forms, with some induced by drugs while others occurring as a chronic progressive disorder associated with aging. Prevalence of osteoporosis can also be attributed to deficiency of calcium and vitamin D and a result of hormonal changes (An et al., 2016).

Bone health largely depends on bone mineral content and density, and bone architecture.

**FIGURE 1:**
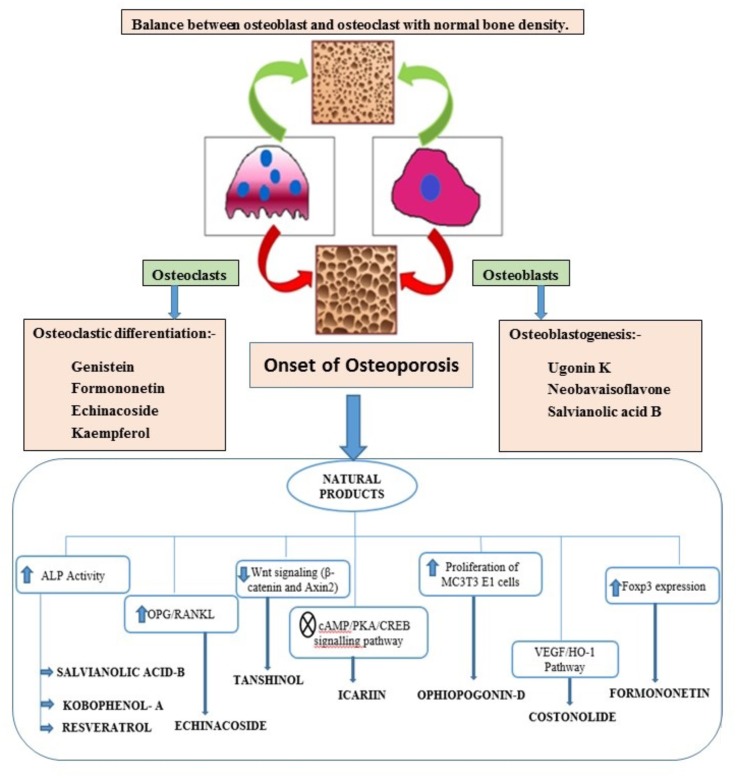
Phytochemicals and their mechanisms intervening various pathways in osteoporosis.

**FIGURE 2:**
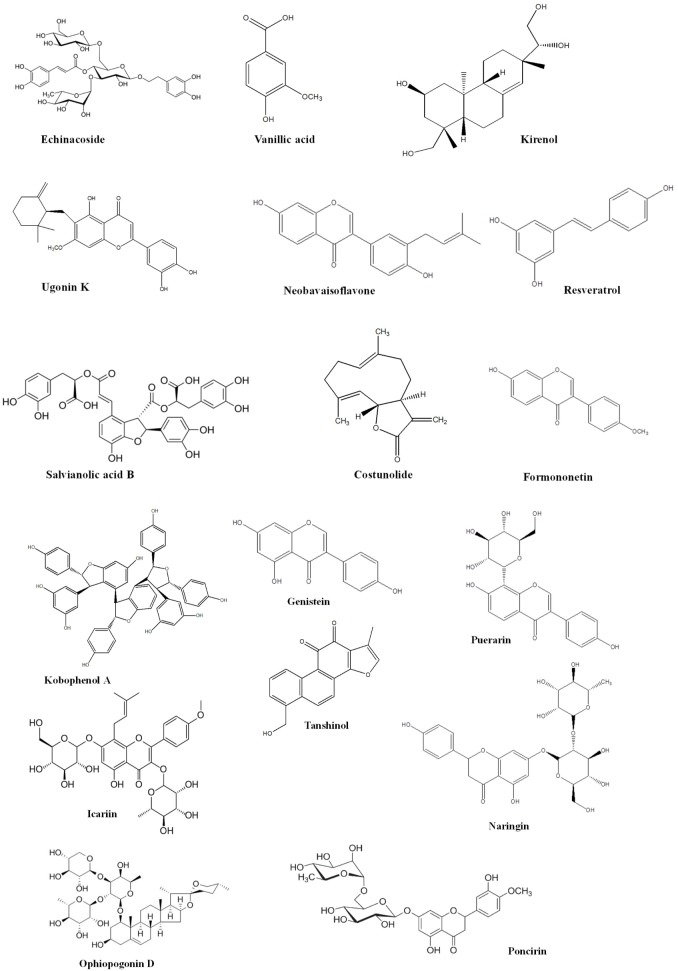
Chemical structures of phytochemicals with antiosteoporotic activity.

Bone is contemplated as a static tissue undergoing constant construction and deconstruction, leading to mineralization of bone matrix and resorption. Synchronization of these processes lead to bone remodeling playing a key role in maintaining normal bone strength, growth, repair, and preservation of calcium homeostasis (Leung and Siu, 2013). Bone modeling and remodeling occur as a result of the action of two types of cells namely osteoblasts, responsible for new bone formation, and osteoclasts, responsible for breakdown of bone. The balance between the actions of these cells is tightly regulated in order to achieve healthy bone formation (Prentice et al., 2006). Osteoblasts accumulate on the bone surfaces resulting the production of the organic matrix, further leading to the extrusion of collagen to form osteoid. This in turn leads to the calcium deposition as amorphous calcium phosphate. The major function of osteoclasts include bone resorption though generation of hydrogen ions leading to solubilization of matrix mineral crystals and providing optimum pH for effective functioning of cysteine protease enzymes. Osteoclastic activity is reported to be stimulated by number of factors including vitamin D, parathyroid hormone (PTH), cortisol, prostaglandins, interleukins, and tumor necrosis factor. Factors comprising of transforming growth factor-β, nitric oxide, estrogen, calcitonin, and androgen are observed to inhibit activity of osteoclasts. Bone diseases like osteoporosis, involving decline of bone density necessitate artificial upregulation of bone mineral density (BMD) with the aid of therapeutic strategies constituting drugs or natural products. These strategies play a key role in maintaining bone mass content and prevention of any uncontrolled decline of the bone loss. Conventional therapies commonly used for the treatment of bone disorders include bisphophates and estrogen Hormonal therapy. But the adverse effects like burning sensation and gastrointestinal tract disturbances associated with these therapies limit their use (Abdel-Naim et al., 2018). Thus the protective effects by natural compounds in multifactorial dysmetabolic disease such as osetoporosis could be a better option to overcome side effects of conventional therapy (Tabatabaei-Malazy et al., 2015). On the other hand osteoarthritis is one of the prevalent joint disease, defined as a heterogeneous group of conditions leading to joint signs and symptoms in turn associated with defective integrity of articular cartilage, in addition to related changes in the underlying bone at the joint margins (Sarzi-Puttini et al., 2005). Osteoarthritis is a chronic progressive disease that has complicated mechanisms that involve inflammation and cartilage degradation. Several phytoconstituents are proven to exhibit beneficial effect in osteoarthritis. The present review summarizes the biological effects of the various phytoconstituents used to maintain the bone health and their associated mechanisms (Putnam et al., 2007). **Figure 1** and **Table 1** represent various phytochemicals and their probable actions of antiosteoporotic activity. **Figure 2** represents chemical structures of phytochemicals reported in this review.

**Table 1:** Chemical and mechanistic profile of phytochemicals.

Sr. No	Chemical classification	Phytoconstituents	Potential mechanism	Reference
1	Phenylpropanoid glycoside	Echinacoside	Increases OPG/RANKL ratio.	Li et al., 2013
2	Phenolic acid	Vanillic acid	Increases BMD and BMC.	Li et al., 2013
		Salvianolic acid B	Upregulates the expression of Runx2, OPN, ALP activity Increases BMP expression, Increases bone mass and diameters. Inhibits the over-production of NO and PGE2, INOS, COX-2, MMP-13, and ADAMTS-5 blocks the IL-1β induced phosphorylation of NF-κB signaling Increases post-fracture ALP activity	Cui et al., 2012 Li and Wang, 2017 Lou et al., 2017 He and Shen, 2014
3	Diterpenoid	Kirenol	Increases OPG/RANKL ratio Increases ALP, ColA1, OPN levels.	Kim et al., 2014
4	Flavonoid	Kaempferol	Increases BMP-2, RUNX-2, osterix, and collagen Inhibits p38, ERK 1/2, and JNK MAP kinase phosphorylation and expression of NFATc1 and c-Fos, enhances expression of Sox9, Runx2, OCN, collagen type I and collagen type X Upregulates the iNOS and Cox-2 expression, Inhibits NF-κB pathway	Kim et al., 2016 Nepal et al., 2013 Zhuang et al., 2017
		Rutin	Enhances the average thickness of trabecular bone Increases ALP activity and vitamin D levels	Zhuang et al., 2017 Abdel-Naim et al., 2018
5	Isoflavone	Neobavaisoflavone	Inhibits apoptosis of osteoblasts Increases Bcl-2 expression.	Chen S. et al., 2017
		Formononetin	Increases FOXP3 expression, Increases bone mechanical properties. Increases expression of VEGF, VEGFR-2/flk-1 ALP, OCN, OPN, and Col I	Mansoori et al., 2016 Huh et al., 2009
		Genistein	Restores bone volume Decreases the RANKL-induced osteoclastic differentiation	Yamaguchi and Levy, 2017
6	Sesquiterpene	Costunolide	Increases VEGF/HO-1 pathways Decreases luciferase activity and Runx2 expression	Lee and Choi, 2011a
7	Stilbene	Kobophenol A	Increases ALP activity Increases Bcl-2 level Decreases Bax expression	Kwak et al., 2013
8	Stilbene	Resveratrol	Decreases serum levels of ALP and OC Increases protein expression of SIRT1 Blocks the expression of TNF-α, IL-1β, IL-6 and IL-18, nitric oxide synthase, nuclear factor (NF)-κB, and reduces caspase-3/9 activity Blocks TLR4 signaling pathway Decreases serum and synovial fluid levels of IL-1β, IL-10, IL-6, TNF-α, MMP-13, and osteocalcin	Kwak et al., 2013 Wei et al., 2018 Jiang et al., 2017 Wang et al., 2016
9	Phytoestrogen	Puerarin	Increases femur trabecular bone structure Involves of ERK1/2 and p38- MAPK pathway	Zhang et al., 2017
10	Prenylated flavonol glycoside	Icariin	Blocks the cAMP/ PKA/ CREB signaling pathway Increases intracellular cAMP levels and PKA and CREB pathways Increases CTX and OC levels and decreases IL-6 levels Decreases phosphorylated p38 and JNK levels, β-catenin levels, MMP-13 levels Inhibits NF-kappaB pathway	Shi et al., 2017 Gao et al., 2017 Zeng et al., 2014 Wei et al., 2017
11	Polyphenol	Tanshinol	Increases FoxO3a and Gadd45a, Decreases canonical Wnt signaling pathway (β-catenin and Axin2)	Yang et al., 2018
12	Steroidal glucoside	Ophiopogonin D	Increases proliferation of MC3T3-E1 cells Decreases TRAP activity Decreases ROS generation	Yang et al., 2018
13	Flavonoid	Ugonin K	Increases proliferation of MC3T3-E1 osteoblasts Increases BSP, OCN levels	Lee et al., 2011 Huang Q. et al., 2015
		Poncirin	Increases bone mineral density, Increases serum OC, Runx2 protein production, expression of OC and OPG mRNA, ALP activity	Lin et al., 2017
		Naringin	Increases BMP-2 expression response via the PI3K, Akt, c-Fos/c-Jun, and AP-1-dependent signaling pathway Increases ALP activity and OCN level Increases the apoptosis of osteoclasts Inhibits RANKL-induced phosphorylation of ERK Reduces synthesis of PGE2, NO, IL-6, and TNF-α	Ang et al., 2011 Wu et al., 2008 Xu et al., 2017

## Echinacoside

Echinacoside (ECH) is most commonly occurring Phenylpropanoid glycoside, from herbaceous flowering and perennial plant, *Echinacea angustifolia* belonging to Asteraceae family. A study exploring effectiveness and safety of ECH, involved the use of osteopenia rat model. On the treatment of overiectomized (OVX) rats for 12 weeks with ECH led to enhancement of osteoprotegerin (OPG) serum levels with concurrent decrease of the receptor activator of NF- κB ligand (RANKL) level (Kohli and Kohli, 2011). This study demonstrated that ECH efficiently and safely prevented OVX induced osteoporosis mediated through increase of OPG/RANKL ratio. The enhancement in the ratio was further proposed to stimulate proliferation of osteoblastic cells, alkaline phosphatase (ALP) activity, COL I contents and OCN levels (Yang et al., 2013). *In vitro* study exploring effects of ECH on osteoblastic MC3T3-E1 cells in the concentration range 0.01 to 10 nM suggested that ECH exhibits potential stimulation of osteoblastic bone formation resulting into promotion of bone regeneration (Li et al., 2013).

An *in vivo* study investigating the effect of ECH in OVX rats at three oral doses including 30, 90, 270 mg/kg for 12 weeks revealed that ECH downsurged the urine concentration of calcium, and inorganic phosphate. Outcomes of Micro- CT analysis of distal femur demonstrated that ECH treatment led to notable enhancement of bone quantity in comparison to OVX groups (Li et al., 2012). Thus study illustrated that antiosteoporotic activity of ECH is mediated through reinforcement of bone formation and downregulation of bone resorption thus validating its role in prevention of osteoporosis (Yang et al., 2013).

## Vanillic Acid

Vanillic acid (VA) is a phenolic acid by chemical nature, abundantly found in *Sambucus williamsii Hance* (SWH). A study exploring the influence of VA on OVX rats following oral administration at a dose of 100 mg/kg body weight illustrated that VA improved BMD and BMC. Further it was found to improve the level of bone turnover markers including calcium, phosphorus, ALP, osteocalcin, deoxypyridinoline (DPD), TNF, and interleukin β and 6. *In vivo* mechanistic study exploring the effect of VA in osteoblasts like UMR-106 cells proved that VA exhibits stimulation of these cells mediated through enhancement of ALP activity and alteration of mRNA expression of genes involved in osteoblastic activity and osteoclastogenesis (Wang Y.G. et al., 2017).

## Kirenol

Kirenol is a diterpenoid, widely isolated from *Herba Siegesbeckiae.* A mechanistic study exploring the effect of kirenol in M3CT3 cells revealed that it promoted differentiation of osteoblasts mediated through signaling pathways like Wnt/β-catenin and BMP and specifically BMP-2. Kirenol was found to augment the expression of markers involved in osteoblasts differentiation like ALP, type I collagen (ColA1), and osteopontin (OPN), low density lipoprotein receptor related protein 5 (LRP5), (OPG/RANKL) ratio, disheveled 2 (DVL2), runt-related transcription factor 2 (Runx2), osterix (Osx), cyclin D1 (CCND1), β-catenin, and phosphorylated glycogen synthase kinase 3β (GSK3β). Moreover Kirenol also led to the enhancement of expression of several factors like ALP, Col A1, CCND1 and β-catenin (Kim et al., 2014).

## Ugonin K

Ugonin K is a flavonoid extracted from the roots of *Helminthostachys zeylanica. In vitro* study in MC3T3-E1 osteoblastic cells demonstrated that Ugonin K greatly stimulated ALP activity and enhanced expression of Bone Sialoprotein (BSP) and OCN. It was found to enhance mineralization of these cell lines and also led to upregulation of osterix, Runx2 expression (Lee et al., 2011). It was shown that enhancement of osteogenesis resulted due to Ugonin K involved a major play by ERK and p38. Moreover Ugonin K was found to greatly induce gene expression for osteocalcin associated with increased ALP activity and BSP expression. Huang Y.L. et al. (2015), in their study illustrated that Ugonin K in combination of estrogen was found to exhibit synergistic, rapid and transient activation of c-Src phosphorylation cascade leading to higher induction of osteogenesis. Ugonin K was found to suppress H_2_O_2_-stimulated reactive oxygen species (ROS) generation. Ugonin K was also observed to offer protection to osteoblastic cells from oxidative stress via regulation of H_2_O_2_ induced ROS generation, activation of ER/Src signaling and downregulation of caspase cascade. Suppression of ROS generation by Ugonin K led to inhibition of apoptotic signaling as evidenced by decreased expression of cytosolic cytochrome *c*, poly (ADP-ribose) polymerase (PARP) and caspase 3, caspase 9 in their active forms. Thus proving potential of Ugonin k in treatment of osteoporosis (Huang Y.L. et al., 2015).

## Neobavaisoflavone

Neobavaisoflavone (NBIF) is a typical isoflavone isolated from *Psoralea corylifolia. In vitro* study exploring osteogenetic activity of NBIF in MC3T3-E1 cells demonstrated that it promotes osteogenesis mediated through upsurge of p38 phosphorylation, ALP activity, and expression of BSP, COL I, OCN, and the mineralization of matrix proteins. Further NBIF was found to stimulate expression of osterix and Runx2 (Don et al., 2012). Moreover NBIF was found to suppress apoptosis of osteoblastic cells leading to inhibition of bone demineralization, a major contributor to osteoporosis. NBIF in combination with psoralen or estradiol was observed to decrease the expression of Bax, p-JNK, IRE1, p-ASK and was also found to upregulate Bcl-2 expression. Thus synergistic regulation of IRE1-ASK1-JNK pathway by the combination of suppressed osteoporotic activity and osteoblastic apoptosis led to greater enhancement of bone mineralization (Chen S. et al., 2017).

## Salvianolic Acid B

Salvianolic acid B (Sal B) is a major phenolic derivative occurring in *Salvia miltiorrhiza.* When tested in human mesenchymal stem cells at concentration of 5 μM, Sal B was found to upregulate expression of ALP activity, Runx2, OPN, and Osx in addition to stimulation of mineralization, mediated through activation of ERK signaling pathways. Further Sal B was observed to augment ERK1/2 phosphorylation in turn leading to enhanced expression of Runx2, an effective contributor of osteogenesis (Cui et al., 2012).

*In vivo* study investigating the influence of Sal B in Gluco-corticoid treated rats at a dose of 40 mg/kg/day for 12 weeks illustrated that Sal B enhanced adipogenesis and precluded gluco-corticoid induced osteopenia. At higher dose of 80 mg/kg/day, Sal B inhibited GC- induced osteopenia in addition to enhanced bone thickness and bone mass, expression of BMPs, peroxisome proliferator-activated receptor (PPARγ), ALP activity, Collagen I protein and microvessel diameters. Sal B was also found to inhibit adipogenesis and AP2 protein expression. A mechanistic study using Sal B in bone marrow stromal cells at a concentration 10^-6^ M to 10^-7^ M was found to augment differentiation of these cells to osteoblast, thus enhanced osteoblastic activity. Further it was observed to downsurge PPARγ mRNA expression accompanied with upregulation of mRNA expression transcribing Runx2 (Li and Wang, 2017). Lou et al. (2017) investigated the anti-inflammatory activity of Sal B in human OA chondrocytes and mouse OA model. *In vitro* study revealed that pretreatment of chondrocytes with Sal B at concentration up to 100 μM for 2 h followed by induction with IL-1β, inhibited the over-production of NO and PGE2 and reversed the elevated expression of INOS, COX-2, MMP-13, and ADAMTS-5. In addition Sal B was found to block the IL-1β induced phosphorylation of NF-κB signaling and p65 nuclear translocation. *In vivo* study using the mouse OA model, induced by destabilization of the medial meniscus showed that Sal B at a dose of 25 mg/kg led to reduction in cartilage degradation proving greater potential of Sal B in OA treatment (Lou et al., 2017). A pilot study using Sprague Dawley rat tibia fracture model treated with Sal B through intraperitoneal route at a dose of 40 mg/kg/d for 3 weeks revealed that treatment with Sal B led to enhancement in callus growth, histological scores and post-fracture ALP activity an indication of promotion of healing process. Thus results suggested that Sal B accelerates early-stage fracture healing and could be developed as a treatment option for trauma fracture patients (He and Shen, 2014).

## Costunolide

Costunolide is a major Sesquiterpene lactone obtained from the roots of *Saussurea lappa.* A study reported by Lee colleagues, revealed that costunolide enhanced cell growth, collagen synthesis and led to upregulation of mineralization of bone. Cell growth enhancement exhibited by costunolide was demonstrated to be mediated through protein kinase C, ERK, mitochondrial ATP-sensitive K^+^ channel and PI3K. Upregulation of collagen synthesis caused by Costunolide was proposed to involve enhanced activation of ER and PI3K. Another study showed that costunolide led to differentiation of osteoblast by upregulating the expression of HO-1, an antioxidant enzyme in mesenchymal stem cells responsible for upregulation of VEGF/HO-1 pathways and Runx2 expression (Jeon et al., 2017). Thus enhancement of HO-1 expression by costunolide in turn leading to enhanced Runx2 expression ultimately led to prevention of osteoporosis (Lee and Choi, 2011b).

## Kobophenol A

Kobophenol A is chemically a tetrameric stilbene, obtained from *Caragana sinica* Rhed. A study involving treatment of human osteoblast like cells with Kobophenol A revealed that Kobophenol A stimulated proliferation of these cells and ALP activity associated with p38 pathway activation through induction of p38 MAPK phosphorylation. Also Kobophenol A treatment led to increased ROS scavenging potential accompanied with upregulation of Bcl-2 and downregulation of Bax expression. Overall these pathways led to enhanced osteoblastic activity, thus supporting the role of Kobophenol A in osteoporotic management (Kwak et al., 2013).

## Formononetin

Formononetin (FNT), an isoflavone extracted from *Butea monosperma* is chemically known as Methoxyisoflavone. A study investigating effect of FNT in OVX female Balb/c mice in combination with PTH as a reference bone healer, demonstrated that PTH significantly upsurged the bone healing property of FNT. Further mechanistic *in vivo* study using OVX mice proved therapeutic potential of FNT, in improving chemistry and biomechanical features of bone thus playing a key role in prevention of osteoporosis progression. Study outcomes revealed, predominant Runx2 and localization of osteocalcin at the site of bone injury (Singh et al., 2017). In addition FNT treatment at a dose of 10 mg/kg through oral route for 4 weeks led to downsurge of Pro-osteoclastogenic subset Th B and Th 17 cells, IL-17A osteoclastogenesis, osteoblastic apoptosis and upregulation of FOXP3 expression. However, FNT administration was found to improve bone chemistry and bone mechanical properties only to a smaller extent (Mansoori et al., 2016). Formononetin when investigated in rat fracture model at a dose of 20 or 200 μg/kg with once a day oral administration during the healing period of 21 days showed that formononetin significantly elevated the number of vessels, vascular endothelial growth factor (VEGF) expression and VEGF receptor 2 (VEGFR-2/flk-1) expression compared with control in the early stage of chondrogenesis. At a higher dose of formononetin no significant increase in expression of VEGF and VEGFR-2/flk-1 was observed. Further at the end of 7 days of administration, formononetin was found to induce significant differentiation of mesenchymal stem cells in the fracture site. After 14 days of administration, a significant stimulation of gene expression of mesenchymal progenitors including ALP, OCN, OPN and Col I, indicating osteogenic differentiation was observed. Thus results indicated that formononetin promotes early fracture healing mediated through activation of angiogenesis in the early stage of fracture repair, and acceleration of osteogenesis in the later stages, and thus could be beneficial for fracture healing (Huh et al., 2009).

## Genistein

Genistein is a widely explored isoflavone extracted from *Genista tinctoria*. Treatment of Preosteoclastic RAW267.4 cells with genistein in combination with alendronate at a concentration of 0.1–100 μM resulted in repression of cell multiplication and cell death. This effect was mediated through suppression of RANKL induced differentiation of osteoclasts (Yamaguchi and Levy, 2017). Further, *in vivo* study exploring synergistic effect of genistein in combination with Silicon and Zinc in OVX Sprague Dawley female rats revealed that administration of genistein in combination with Silicon for a duration of 10 weeks exhibited maximum BMD of femur and lumbar spine, thus restoring bone volume and thickness of femoral trabecular bone. Moreover genistein and silicon combination showed the highest upsurge in phosphorus and calcium levels in serum of OVX rats, compared to stand alone treatment with genistein or silicon (Qi and Zheng, 2017). Genistein was observed to prevent bone loss through induction of osteoblast differentiation mediated through upregulation of the Wnt/β-catenin, Runx2, PPARγ, BMP2, estrogen and Smad5 in Dai et al. (2013) and inhibition of various transcription factors such as cytokinesTGF-β, IGF-1, and NF-κB (Joo et al., 2004). Genistein treatment in femurs of ob/ob mice, a model for obesity and type two diabetes mellitus revealed that genistein improves resistance to fracture from bending loads. Genistein treatment increased ultimate force required to fracture the femur and the maximum deformation to failure (Odle et al., 2017).

## Resveratrol

Resveratrol is chemically a Stilbene, abundantly occurring in red grapes. An *in vivo* study evaluating effect of Resveratrol in osteoporotic rat model showed that Resveratrol significantly increased BMD, femoral porosity and biomechanical property. Further Resveratrol treatment was demonstrated to downregulate OC and ALP activity and enhanced expression of SIRT-1 protein and IkBα protein, in addition to inhibition of NF-κB (Wang X. et al., 2017). Moreover, it was also found to decline RANKL, Tartrate-resistant acid phosphatase-5b (TRAP-5b) level in addition to upsurge of OPG level and inhibition of damage of bone microarchitecture. Inhibition of RANKL by resveratrol was attributed to its antioxidant activity which in turn led to inhibition of osteoclastogenesis in the OVX rats. *In vitro* study using osteoclasts revealed that RES inhibited expression of mRNA transcripting osteoclast specific enzyme mediated through improvement of oxidative stress damage of osteoclast. Thus Resveratrol was proved to offer significant bone protection through suppression of function and multiplication of osteoclasts as illustrated by *in vivo and in vitro* studies. Further a preclinical study using osteoporosis mice model showed that RES significantly precluded bone loss and reversed the decrease of type 1 collagen, Runx2, OCN in addition to upregulation of FOXO1 level. Mechanistic *in vitro* study using MC3T3-E1 cells demonstrated that RES lowers OPG/RANKL ratio leading to subsequent inhibition of osteoclastogenesis. Another *in vitro* study using human osteoblast (HOB) cells revealed that RES suppresses miR-338-3p leading to subsequent increase in the Runx2 expression (Zhao et al., 2015). The effectiveness of resveratrol was examined in a rat model of osteoarthritis wherein it was observed to improve inflammatory damage and offer protection against OA via NF-κB and HO-1/Nrf-2 signaling. Resveratrol was found to block the induction of clinical scores, expression of tumor necrosis factor-α, interleukin (IL)-1β, IL-6 and IL-18, and reduced caspase-3/9 activity in rats with OA. In addition it was observed to supress expression of inducible nitric oxide synthase (iNOS), nuclear factor (NF)-κB, phosphorylated-(p)-AMP-activated protein kinase and sirtuin 1 protein and stimulate expression of heme oxygenase 1 (HO-1) and nuclear factor erythroid 2-related factor 2 (Nrf-2) protein (Wei et al., 2018).

A study carried out to evaluate the effect oral resveratrol on obesity-related OA developed with C57BL/6J mice using high-fat diet involved 12 weeks administration of two doses (22.5 and 45 mg/kg) of resveratrol by gavage. Study outcomes revealed that resveratrol at 45 mg/kg significantly improved OA symptoms and reduced levels of serum IL-1β and leptin. In addition resveratrol was found to significantly inhibit the expression of TLR4 and TRAF6 in cartilage. Thus, it was concluded from the study that resveratrol could be an effective option for improvement of OA pathology by reducing the levels of inflammation and/or blocking TLR4 signaling pathway in cartilage (Jiang et al., 2017). Moreover a study to evaluate the protective effects of resveratrol on monosodium iodoacetate (MIA)-induced OA through inhibition of cyclooxygenase (COX-2) and iNOS signaling pathway in a rat model involved injection of MIA followed by treatment with resveratrol at a dose of either 5 or 10 mg/kg body weight. The results revealed that resveratrol significantly decreased hyperalgesia of mechanical, heat and cold and increased the vertical and horizontal movements. In addition resveratrol was found to decrease serum and synovial fluid levels of IL-1β, IL-10, IL-6, TNF-α, MMP-13 and osteoclastic activity marker, osteocalcin and its articular cartilage mRNA and protein expressions induced by MIA treatment. The protection offered by resveratrol was comparable to a reference drug, etoricoxib. Resveratrol was also observed to attenuate the cartilage damage induced by MIA. Thus resveratrol was proven to improve MIA-induced cartilage damage by inhibiting the levels and expressions of inflammatory mediators, which strongly suggested resveratrol to be a potential therapeutic agent for OA (Wang et al., 2016). On the other hand another study revealed that long term treatment with high-dose RSV reduced bone mass and fracture strength and did not prevent immobilization-induced bone loss in rats. RSV also decreased femoral mid-diaphyseal three-point bending strength and stiffness and it was unable to prevent significant bone loss and reduced bone strength induced by BTX (Ornstrup et al., 2018).

## Puerarin

Puerarin is a widely explored phytoestrogen found in *Pueraria lobata (Willd.)* Ohwi. It is investigated for its bone loss prevention and bone regeneration enhancement property. *In vitro* study investigating effect of Puerarin on Bone Marrow stromal cells showed that Puerarin promotes osteogenin differentiation in these cells. The probable mechanism proposed was involvement of Puerarin in MAPK signaling pathway and ERK1/2 -Runx2 signaling pathway (Zhang et al., 2017). *In vivo* study conducted using OVX rats to evaluate antiosteoporotic action of puerarin showed that puerarin offered protection against decline of BMD and also improved structure of trabecular bone. Another preclinical study in OVX rats exploring synergism between Puerarin and Zinc revealed that this combination additively prevented mandibular bone loss mediated through inhibition of osteoclastogenesis. Other outcomes involved decrease in BMD, bone morphological features, upregulation of OPG, OPN, Ca^++^ levels and downregulation of TRAP and RANKL levels. Further, coadministration of Puerarin and Zinc was found to reverse OVX stimulated bone loss and decrease bone marrow adiposity in treated rats (Liu et al., 2016).

## Icariin

Icariin is a commonly occurring prenylated flavonoid glycoside obtained from *Herba epimedium*. An *in vitro* study exploring the effect of Icariin on bone marrow cells revealed that Icariin suppressed differentiation and bone resorption activity of osteoclast and enhanced osteogenic differentiation. Mechanistic *in vitro* study in osteoblastic cells revealed that Icariin suppressed osteogenic activity through blockade of cAMP/PKA/CREB signaling pathway, known to be an inhibitor of cAMP – synthesizing Adenylyl cyclase and Protein kinase A. These effects were brought about by enhancement of intracellular cAMP levels and phosphorylation of cAMP response element binding (CREB) and PKA (Shi et al., 2017). Further exploration revealed that mechanism involved upregulation of peak bone mass, increased mineralization, and induction of growth maturation of osteoblasts. A study exploring synergism between Icariin and Adipose- derived stem cell transplantation in osteoporotic rats illustrated that the combination enhanced the serum levels of TRAP, ALP activity, OPG and bone γ-carboxyglutamate protein (Tang et al., 2018). Further, the treatment was found to significantly reduce body weight gain and elevate BMD of lumbar spine, femur bone, in addition to improvement in biomechanical properties of femur. A study investigating the effect of icariin in mouse osteoarthritis (OA) model revealed that ICA offers protection to subchondral bone, hyaline, and calcified cartilage. ICA was found to improve bone remodeling in subchondral bone of OA. Early intervention of ICA on OA is proven to be more effective. Icariin had positive influence on serum bone turnover markers expressions and histological changes of cartilage when tested at a dose of 10 mg/(kg⋅day). Icariin treatment led to significant increase in the levels of CTX and OC and decreased the levels of IL-6, and OARSI scores. Histological studies showed that the tibial cartilage loss was significantly improved with icariin treatment (Gao et al., 2017).

Matrix metalloproteinase-13 (MMP-13) is an enzyme when present in excess plays a critical role in the cartilage breakdown. Therefore, the effects of Icariin on the expression of MMP-13 in IL-1β-induced SW 1353 chondrosarcoma cells was investigated followed by the study of *in vivo* effects of Icariin on an experimental rat model of OA induced by anterior cruciate ligament transection (ACLT). The results revealed that treatment with Icariin decreased the number of cartilage lesions, phosphorylated p38 and JNK levels, β-catenin levels in both IL-1β-induced SW1353 chondrosarcoma cells and in the rat OA model (Wang Z. et al., 2017). Surprisingly, the MMP-13 inhibition offered by Icariin on was found to be significant than exhibited by other signaling pathway inhibitors. Thus the study proposed Icariin to possess therapeutic potential for the treatment of OA (Zeng et al., 2014). Further *in vitro* study using chondrocytes revealed that icariin offers protection from damage through reduction of the NF-kappaB (P65) activities and enhancing the expression of IkappaBalpha by chondrocytes (Wei et al., 2017).

## Tanshinol

Tanshinol is abundantly found polyphenol from roots of *Salvia miltiorrhiza Bunge.* An *in vivo* study using glucocorticoids induced bone loss rat model revealed that tanshinol treatment for a duration of 14 weeks resulted in improvement of biomechanical property as shown by micro- CT analysis. Mechanistic exploration showed that Tanshinol upregulated FoxO3a and Gadd45a proteins involved in FoxO3a pathway and suppressed Axin2 and β-catenin involved in canonical Wnt signaling pathway in turn leading to enhancement of osteogenesis. Thus, Tanshinol was shown to counteract impairment of bone formation through KLF15/PPARγ2/FoxO3a/Wnt pathway and abnormal expression of signaling molecules caused by glucocorticoids thus effectively stimulating osteogenesis (Yang et al., 2018). Coadministration of Tanshinol with Calcitriol in GC-induced bone loss model was found to improve bone microarchitecture and inhibit bone loss induced by glucocorticoids through promotion of bone generation and downsurge of bone resorption (Chen G. et al., 2017).

## Ophiopogonin D

Ophiopogonin D is a Steroidal glucoside obtained from perennial plant named as o*phiopogon japonicus.* Its antiosteoporotic activity is attributed to antioxidant activity leading to reduction in oxidative stress. Treatment of MC3T3-E1 mouse pre-osteoblast cell line and RAW264.7 macrophage cell line with Ophiopogonin D demonstrated that treatment reduced ROS generation mediated through FoxO3a-β-catenin signaling pathway, and decreased levels of bone degradation markers like CTX-1 and TRAP (Huang Q. et al., 2015).

## Naringin

Naringin is a polymethoxylated flavonoid isolated from Grapefruit. When tested in primary cultured osteoblast, Naringin was found to enhance BMP-2 expression and osteogenic response mediated through Akt, PI3K, c-Fos/c-Jun, and AP-1. Further it was found to increase ALP activity, OPN synthesis, cell proliferation, and OCN level. BMP-2 transcriptional regulation was mediated through Akt phosphorylation and c-Jun and c-Fos activator protein (AP-1) components. Naringin was found to stimulate binding of c-Jun and c-Fos to the AP-1 protein associated with BMP-2 promoter (Wu et al., 2008). Moreover, *in vitro* studies using osteoclasts revealed that naringin enhanced apoptosis of osteoclasts. In addition Naringin was found to suppress expression of key marker genes of osteoclast in turn leading to inhibition of RANKL induced NF-κb activation through suppression of RANKL mediated IκB-α degradation. Naringin was also found to inhibit RANKL induced ERK phosphorylation (Li et al., 2014). Further *in vivo* study showed that naringin improved BMD, thickness of trabecular bone, bone mineralization and bone mechanical strength. Further it was proven to downregulate Bcl-2 mRNA expression and upregulate caspase -3, Bax, and Cyt c levels. Furthermore, naringin was shown to significantly decrease bone resorption area and enhance apoptosis of osteoclast, mediated through regulation of mitochondria apoptosis pathway. Overall these studies revealed that naringin has beneficial effect in the prevention and treatment of osteoporosis through inhibition of osteoclast formation and bone resorption (Ang et al., 2011). A study to investigate the anti-osteoarthritic and anti-inflammatory effect of naringin in MIA- induced osteoarthritis (OA) rat model revealed that naringin promoted the recovery of hind-limb weight-bearing, reduced the generation or production of inflammatory mediator and proinflammatory cytokines, and protected the tissue from the damage. *In vitro* study in lipopolysaccharide-induced RAW 264.6 cells revealed that naringin exerted an anti-inflammatory effect through reduction of the production of the prostaglandin E2 (PGE2), nitric oxide (NO), interlukin-6 (IL-6), and tumor necrosis factor-α (TNF-α) in LPS-induced RAW cells. Thus the outcomes revealed naringin to be an effective therapeutic drug for the treatment of the OA and OA-related symptoms (Xu et al., 2017). An *in vitro* study conducted to evaluate the role of naringin in cartilage degeneration involved use of primary murine chondrocytes cultured with stimulation of TNF-α, in the presence or absence of naringin treatment. Naringin was found to attenuate TNF-α-mediated inflammation and catabolism in chondrocytes. The study involving surgically induced OA mice models showed that oral administration of naringin improved degradation of cartilage matrix and offered protection against OA development. Moreover the protective effect of naringin in cartilage and chondrocyte was attributed to suppression of NF-κB signaling pathway (Zhao et al., 2016).

## Poncirin

Poncirin is a flavonoid obtained from the fruit of *Poncirus trifoliata.* It is widely explored antiosteoporotic drug involved in regulation OPG/RANKL ratio, extracellular-signal-regulated kinase/c-Jun N terminal kinase/mitogen-activated protein kinase (ERK/JNK/MAPK), estrogen receptor (ER), bone morphogenetic protein (BMP), transforming growth factor (TGF)-β, Wnt/β-catenin, and Notch signaling pathways (Lin et al., 2017). Poncirin when tested in GC induced mice revealed that treatment resulted in enhancement of OC, OPG, mineral nodule formation and mRNA expression and decrease in CTX serum level. Poncirin was also found to significantly increase BMD and improve bone microarchitecture (Yoon et al., 2012).

## Kaempferol

Kaempferol is a flavonoid derived from the rhizome of *Kaempferia galanga L*. Mechanistic study revealed that osteogenic differentiation caused by Kaempferol led to mineralization and increased osteogenic gene expression. At a concentration up to 10 μM when tested in osteoblast, Kaempferol was found to increase osteoblastic activated factor including BMP-2, RUNX-2, osterix, and collagen (Kim et al., 2016). Another study revealed that Kaempferol suppressed osteoclast differentiation mediated through activation of IL-1β and RANKL. Kaempferol also led to prevention of osteoclast formation in turn leading to prevention of bone loss (Lee et al., 2014). Kaempferol was found to inhibit p38, ERK 1/2, and JNK MAP kinase phosphorylation and expression of NFATc1 and c-Fos ultimately leading to prevention of osteoclast formation. *In vitro* study involving clonal mouse chondrogenic ATDC5 cells revealed that Kaempferol treatment induced chondrogenic differentiation and clustering of cartilage nodules. In addition kaempferol treatment led to increased synthesis of matrix proteoglycans and enhanced expression of chondriogenic marker genes including Sox9, Runx2, OCN, collagen type I and collagen type X. Further kaempferol treatment resulted in increase in ALP activity and selective induction of extracellular signal regulated kinase (ERK) over p38 MAP kinase or c-jun N-terminal kinase. Thus Kaempferol turned out to be a potential candidate for treatment of all bone development disorder (Nepal et al., 2013). A study designed to examine the anti-inflammatory and anti-osteoarthritis (OA) effects of kaempferol in rat articular chondrocytes revealed that Kaempferol treatment (up to 100 μM) resulted in the reduction in the interleukin-1b-stimulated formations of PGE2 and NO. Kaempferol was found to upregulate the iNOS and Cox-2 expression in interleukin-1β-stimulated rat OA chondrocytes. In addition Kaempferol significantly reduced in interleukin-1β-stimulated pro-inflammatory mediators in rat OA chondrocytes by inhibiting the NF-κB pathway. Thus results suggested that kaempferol had significant anti-inflammatory and anti-arthritis effects proposing kaempferol to be a novel therapeutic active agent in OA management (Zhuang et al., 2017).

## Rutin

Rutin is a flavonoid known to have a variety of biological activities including antiallergic, anti-inflammatory, antiproliferative, and anticarcinogenic properties. A study to explore the anti-osteoporotic effect of rutin (quercetin-3-*O*-rutinose) on ovariectomized female rats revealed that Intragastric administration of rutin once daily for 3 months resulted in significant improvement of the BMD and decreased the level of inflammatory factors like IL-6, TNF-α, and INF-γ in OVX rats. In addition, rutin was found to significantly enhance the average thickness of trabecular bone and the average bone volume fraction substantiating use of Rutin in treatment of osteoporosis (Wang Q.L. et al., 2017). An *in vitro* study using SAOS-2 cells revealed that rutin increased osteocyte and osteoblast-related gene expression and reduced the expression of RUNX suppressor and osteoclast genes. Further rutin was found to increase ALP activity and vitamin D levels and decreased acid phosphatase which is a marker of osteoporosis. Thus, study concluded that rutin increased proliferation and ossification markers in bone cells (Abdel-Naim et al., 2018).

In another study rutin was found to induce bone formation mediated through the differentiation of MG-63 cells associated with increased ALP activity (Hyun et al., 2014). Furthermore a study conducted to evaluate the effect of rutin on bone metabolism in ovariectomized (OVX) rats revealed that rutin prevented reduction in both total and distal metaphyseal femoral mineral density caused by ovariectomy (Horcajada et al., 2015). Rutin enhanced femoral failure load, plasma osteocalcin (OC) concentration (a marker for osteoblastic activity) and reduced urinary deoxypyridinoline (DPD) excretion (a marker for bone resorption) and calciuria. Thus the outcomes of the study revealed that rutin and its metabolites inhibit ovariectomy-induced trabecular bone loss in rats, by slowing down resorption and enhancing osteoblastic activity (Horcajada-Molteni et al., 2000).

## Conclusion

Bone disorders majorly including osteoporosis and osteoarthrtis are becoming prevalent with the growth in the aging population. There are only few conventional therapies available for the treatment and prevention of osteoporosis which include natural and synthetic estrogens, hormone replacement therapy, calcium in combination with vitamin D and bisphosphonates. Unfortunately, all of these therapies suffer from significant drawbacks. The adverse effects like burning sensation and gastrointestinal tract disturbances associated with these therapies limit their use (Abdel-Naim et al., 2018). Thus the protective effects by natural compounds in multifactorial dysmetabolic disease such as osetoporosis could be an effective alternative to overcome side effects of conventional therapy. Osteoporosis could be better treated through a multitarget therapy including the association of multiple herbal drugs and natural compounds (Tabatabaei-Malazy et al., 2015; Menghini et al., 2016). This review proved that the supplementation of phytochemicals play a key role in prevention of osteoporosis, leading to prevention of the bone loss and demineralization. These effects were reported to be mediated through several mechanisms including upregulation the expression of various factors like Runx2, OPN, and Osx in addition to upregulation of pathways like MAPK pathway, OPG/RANKL ratio and VEGF/HO-1 pathway etc. Diverse mechanisms exhibited by each phytochemical proved that every molecule has specific interaction with target pathways and enzymes irrespective of its chemical structure. Various phytochemicals exhibited similar mechanistic property irrespective of their chemical diversity. Further phytochemicals were found to exhibit multiple beneficial activities which include decreased bone resorption, enhanced osteoblastic activity and downregulation of the osteoclastic activity. Several phytochemicals when explored in combination with standard therapies were illustrated to produce synergistic effects. Phytochemicals majorly including flavonoids have been proved to have beneficial effects on joint health through reduction of inflammation in animal models of arthritis and promoting crosslinking of collagen (Curtis et al., 2000). Thus this review offers phytochemicals as an aid to combat osteoporosis and osteoarthritis in conjunction with available therapies and to prevent bone disorders.

## Author Contributions

VS contributed toward conceptualization, planning, and writing the paper. MS, TK, and PC contributed toward data collection and writing the paper. AS, KP, and BD contributed toward editing of the manuscript.

## Conflict of Interest Statement

The authors declare that the research was conducted in the absence of any commercial or financial relationships that could be construed as a potential conflict of interest.
